# Preparation and Characterization of Water-Based Nano-Perfumes

**DOI:** 10.3390/nano8120981

**Published:** 2018-11-27

**Authors:** Małgorzata Miastkowska, Elwira Lasoń, Elżbieta Sikora, Katarzyna Wolińska-Kennard

**Affiliations:** 1 Institute of Organic Chemistry and Technology, Cracow University of Technology, Warszawska 24, 31-155 Krakow, Poland; elason@chemia.pk.edu.pl (E.L.); esikora@pk.edu.pl (E.S.); 2 Finea Sp. Z o.o., Sternicza 129/55, 01-350 Warsaw, Poland; katarzyna.wolinska@yahoo.com

**Keywords:** fragrances, nano-perfumes, nanoemulsions

## Abstract

The application of nanoemulsions as a novel delivery system for lipophilic materials, such as essential oils, flavors, and fragrances is one of the growing technologies used in cosmetic, pharmaceutical, and food industries. Their characteristic properties, like small droplet size with high interfacial area, transparent or semi-transparent appearance, low viscosity, and high kinetic stability, make them a perfect vehicle for fragrances, in the perfume industry. They could be a great alternative to water-based perfumes, without alcohol, and solve problems related to the oxidation and low bioavailability of fragrances with other non-alcoholic vehicles of perfumes like pomades or gels. The aim of our study was to develop stable Oil-in-Water (O/W) nanoemulsions that are compatible with selected fragrance compositions, without ethanol, polyols, and ionic surfactants, and to study their physicochemical, microbiological, and dermatological properties. The nano-perfume systems were obtained with a low-energy (Phase Inversion Composition; PIC) and with a high-energy (ultrasound, US) method, taking into account the possibility of moving from the laboratory scale to an industrial scale. The optimized nano-perfume formulations, prepared with different methods, yielded the same physicochemical properties (stability, medium droplet size of the inner phase, polydispersity, viscosity, surface tension, pH, density). Stable systems were obtained with a fragrance composition concentration within 6–15% range. These formulations had a low viscosity and a pH suitable for the skin. Moreover, the obtained results confirmed the protective role of nanoemulsions. The peroxide number measurement (POV) showed that the tested fragrance compositions had a high chemical stability. The results of the microbiological tests confirmed that the obtained products were free of microbiological contamination and were appropriately preserved. The dermatological test results confirmed the safety of the developed preparations.

## 1. Introduction

Ethanol forms the base for traditional perfumes available on the market, since it is the solvent of hydrophobic aromatic substances. Depending on the contents of a fragrance composition, we distinguish between eau de parfum which contains 10–15% of a fragrance composition, eau de toilette which contains 5–10% of a fragrance composition, and eau de cologne which contains 3–5% of a fragrance composition [1,2]. Unfortunately, people prone to allergies or with sensitive skin, who use ethanol-based perfumes, may suffer from skin irritation and inflammation because ethanol is a solvent with a defined irritating potential [3].

As an alternative to alcohol compositions, there are perfumes in a solid state (solid emulsions, gels, and pomades) and perfumed oils. However, their main disadvantage is that they leave greasy and slippery spots on the perfumed surfaces. Some examples of perfumed products are presented in the literature and they are based on a safe solvent like water, the so-called “alcohol-free perfumes” (emulsions, microemulsions, liposomes, and micelles) [4,5,6,7,8,9]. Yet from a technological perspective, the introduction of lipophilic systems to water without a cosurfactant (ethanol, among others) is very difficult, with respect to thermodynamic stability. It requires the use of solubilizers of essential oils which can be polyols (glycols and glycerin) or surfactants.

An interesting solution for the water-based perfumes without alcohol, seem to be nanoemulsions—liquid colloidal systems characterized by a high degree of dispersion (20–500 nm), consisting of aqueous and oil phases and a surfactant, sometimes with the addition of a cosurfactant. Similar to microemulsions, nanoemulsions increase the expiry date of many products, due to their resistance to sedimentation and creaming. An additional advantage over microemulsions is that they have a much lower amount of a surfactant (approx. 5–10%), which allows maintaining adequate stability of the system and it makes them safe for human body [10,11,12,13,14]. Clear appearance, liquidity, and low viscosity, make nanoemulsions even more popular with the cosmetic industry. The examples of nanoemulsion cosmetics found on the market are anti-UV hair spray by Korres, Nanocream by Sinerga, Nanogel by Kemira, Vital Nanoemulsion A-VC serum by Marie Louise, Bepanthol Ultra face cream by Bayer, or face cleanser NanoVital by Vitacos Cosmetics [15,16]. Moreover, the international cosmetics company L’Oreal patented a number of formulas for nanoemulsion cosmetics [15,16]. In the case of perfume products, nanoemulsions can be not only a medium for a fragrance composition, but they also increase chemical stability of the compounds in the composition (protection against oxidation) [12,13,17,18].

Alcohol-free perfumes based on micro- and nanoemulsions are already present in the patent literature [19,20,21,22], however, apart from additional solubilizers, such as polyols or paraffin hydrocarbons, the compositions of the perfumes in the above-mentioned patents have cationic or anionic surfactants, which can be an irritant to the skin. Nanoemulsions that serve as a matrix for fragrance substances used in cosmetics are also described in the literature [18,23,24,25,26,27,28] these are primarily solutions for emulsification of a single lipophilic component that forms the oil phase of a nanoemulsion (e.g., D-limonene) [18,23,24]. On the other hand, the fragrance compositions contain up to twenty compounds with various chemical structures (alcohols, phenols, aldehydes, esters, saturated, unsaturated, cyclic, and branched hydrocarbons), and as a result they are a difficult base for obtaining stable nanoemulsion systems. 

Nanoemulsions turned out to be a great solution for solving problems related to the oxidation and low-bioavailability of fragrances and they could be applied as nano-encapsulated fragrance systems, in the perfume industry. Therefore, the most important aspect for further research would be to make the preparation of fragrance nanoemulsions practicable, at a pilot scale, so as to make them possible to be adopted in the industrial full-scale production.

The aim of the research was to develop stable oil-in-water (O/W) nanoemulsions that are compatible with selected fragrance compositions, without ethanol, polyols, and ionic surfactants, and to study the physicochemical, microbiological, and dermatological properties, as well as permanence of the fragrance of the obtained nano-perfumes. The nano-perfume systems were obtained with a low-energy method (Phase Inversion Composition; PIC), as well as with an ultrasound (US) high-energy method, taking into account the possibility of moving from the laboratory scale to an industrial scale.

## 2. Materials and Methods

### 2.1. The Properties of Raw Materials

In order to obtain dispersive systems with the droplet size of 20–500 nm of the inner phase, the research concerned the influence of the type and concentration of the oil phase (of a given fragrance composition) and of the type and concentration of the surfactant on the stability of nanoemulsion systems. The emulsifiers used in the study were supplied by the Croda company (Krakow, Poland) (Table 1). Fragrance compositions were made by European Flavours & Fragrances PLC (Hertfordshire, UK). All tested fragrances are of GRAS (Generally Recognised As Safe) status, allergen free. Milli-Q® filtered water (Merck, Warsaw, Poland) was used as the aqueous phase of the nanoemulsions. The preservative used was Dermosoft 1388 (up to 1%), kindly supplied by Evonik Dr. Straetmans GmbH (Germany).

Nonionic surface active agents were used in the research. Those surfactants belong to the group of polyoxyethylated esters of glycerin and fatty acids, polyoxyethylated castor oil, polyglycerol and fatty acids esters, and alkyl polyglucosides. Surfactants of this type are known for their very good performance and dermatological properties. They show biocompatibility with the skin and they are used in cosmetics, such as solubilizers, humectants, dispersing agents, and emulsifiers, for the stabilization of O/W emulsions. In comparison to the ionic surfactants, both cationic and anionic, they are not susceptible to pH change and the addition of electrolytes. They can be used with other emulsifiers to increase the system stability [13,29,30,31].

### 2.2. The Method of Obtaining Nanoemulsions

#### 2.2.1. Phase Inversion Composition Method (PIC)

To obtain perfumes in the form of a nanoemulsion, the low-energy method was used (Phase Inversion Composition—PIC). The nanoemulsions were obtained by a gradual addition of water to the mixture of a surfactant with the oil phase (the fragrance composition), at room temperature (25 °C), with constant stirring (IKA Vortex Genius 3 shaker).

#### 2.2.2. Ultrasonic Homogenization Method (US)

The nano-perfumes in the nanoemulsion form were obtained with a high-energy method that requires ultrasonic homogenization with initial pre-emulsification. A specified amount of the oil phase (fragrance composition), surfactant, and demineralized water was dispersed at room temperature (25 °C), with a mechanical stirrer (IKA° RW 20 digital), 500 rpm, for 10 min. The obtained pre-emulsion underwent ultrasonic homogenization (probe-type sonicator UP200Ht, Hielscher Company, Teltow, Germany) with 15 W for at least 60 s.

### 2.3. Tests of the Physicochemical Properties of the Fragrance Compositions

In order to establish the properties of the fragrance compositions used in the research, their surface tension was measured with a tensiometer STA-1 by Sinterface (Berlin, Germany) with a du Noüy ring. Viscosity was determined with an R/S (Cone/Plate) rotational rheometer with cone/plate measuring elements (by Brookfield). Moreover, the value of logP was calculated (i.e., lipophilicity of the composition) on the basis of the qualitative and quantitative constituents of the used fragrance compositions provided in material data sheets. This was determined by calculating the lipophilicity (logP, P—Partition coefficient) of individual relevant constituents and the percentage composition of the fragrance [32].

### 2.4. Tests of the Physicochemical Properties of the Nanoemulsion

In order to determine the physicochemical properties of the obtained systems, the following analytical methods and techniques were used. The size of the droplets of the inner phase was analyzed with a Zetasizer Nano ZS droplet analyzer (Malvern Instruments, Malvern, UK). The kinetic stability of the emulsion systems was monitored with the analysis of the droplet size of the inner phase of the nanoemulsion and the polydispersity index (PDI) over time. To determine the rheological properties of the obtained formulations, just like in the case of pure compositions, an R/S rotational rheometer was used with cone/plate measuring elements (cone C25-1), at room temperature (25 °C). Viscosity tests were conducted with a variable cutting rate, within the range of 1–500 rps. The surface tension of the emulsion was also measured with a tensiometer and a du Noüy ring, in the same way as in the case of the fragrance compositions. The pH value of the emulsion system was determined with a multi-functional measuring tool (Seven Multi by Mettler Toledo, Warsaw, Poland) which was equipped with an electrode for measuring pH. Density was established with a hydrostatic method, using an analytical balance (AS310X0, Radwag, Krakow, Poland), with a density measuring device.

### 2.5. The Test of the Microbiological Properties of the Nanoemulsion

The test of microbiological purity of the obtained stable products was conducted according to the following polish standards: PN-EN ISO 21149:2009, PN-EN ISO 162012:2011, PN-EN ISO 22718:2010, PN-EN ISO 21150:2010, and PN-EN ISO 18416:2009. The preservation tests were carried out according to the standard PN-EN ISO 11930:2012. The following sample strains were used: *Pseudomonas aeruginosa* ATCC 9027, *Staphylococcus aureus* ATCC 6538, *Escherichia coli* ATCC 11229, *Candida albicans* ATCC 10231, and *Aspergillus brasilensis* ATCC. With regard to every checked microorganism, the test involved the treatment of a cosmetic preparation with a standardized inoculum and then the measurement of the number change of the microorganisms, at given intervals, during a specified period and under a given temperature. 

### 2.6. The Test of the Dermatological Properties of the Nanoemulsion

The dermatological test of the obtained products was conducted with a patch test on a group of twenty-five testers (15 women and 10 men, age 19–55), under a dermatologist’s supervision, in accordance with the applicable legal provisions and guidelines for dermatological research on humans. The tests were conducted in a specialist dermatological medical room. Patch tests were applied on the inner side of the forearm. The tested preparation was rubbed onto a 1 × 1 cm skin patch, the area with an applied cosmetic was covered with a 2 × 2 cm filter paper and foil attached with an adhesive. The dressing was taken off after 24 h and the result was assessed organoleptically. Further dermatological check was carried out after 3, 4, and 5 days, after application. The assessment was conducted according to the generally accepted scale for dermatological tests [33].

### 2.7. The Test of Fragrance Permanence of the Nanoemulsion

The test of fragrance permanence was carried out in accordance with the standard BN-84/6148-02. Two strips of filter paper, 150 mm long and 15–20 mm broad, were dipped 20 mm in the samples; the first one was dipped in a tested sample, and the second one in a reference sample, and left at the temperature of 25 °C. The intensity of the fragrance was assessed organoleptically after 1, 24, and 48 h after dipping. The samples were assessed by comparing the intensity of the fragrance of the tested sample and the reference sample. 

### 2.8. The Test of Chemical Stability of the Fragrance Composition (Oxidative Stress Test)

The oxidative stress test was carried out to demonstrate the chemical stability of the fragrances and protecting effect of nanoemulsions against peroxidation of the fragrance composition. The oxidation process induced by the oxygen from air and by UV, was determined by measuring the peroxide value of the fragrances, according to Wheeler DGF standard method C-VI 6a [34]. The value was measured as the amount of iodine which was formed by the reaction of peroxides (formed in oil) with iodide ion. The sample was added to a mixture of glacial acetic acid and isooctane (60/40 *v*/*v*) and then allowed to react with potassium iodide (0.5 cm^3^ of saturated solution). The iodine released was determined by titration, using 0.01 N sodium thiosulfate solution. The titration end point was specified iodometrically. The peroxide value (POV) was calculated according to Equation (1):

(1) $$POV=\frac{\left(a-b\right)\times M\times 1000}{2\times Q}$$

where *a* is the consumed volume of sodium thiosulfate solution, *b* is consumed volume of sodium thiosulfate solution in the blank test, *M* is the molarity of the sodium thiosulfate solution, and *Q* is the quantity of the tested sample with accuracy ±0.1 mg. Each sample was assessed in triplicates (*n* = 3).

### 2.9. Statistical Analysis

All data presented in the plots were presented as a mean of three different experiments ± SD. Differences between the calculated means of each individual group were determined by one-way ANOVA tests, using the statistical software Statistica Version 12 StatSoft Company., Cracow, Poland. A value of *p* < 0.05 was considered statistically significant.

## 3. Results and Discussion

### 3.1. The Properties of the Tested Fragrance Compositions

In order to establish the properties of a fragrance composition, their logP was calculated and the surface tension (τ) and viscosity coefficient (η) were tested.

As can be seen from Table 2, composition B had the highest logP, surface tension, and viscosity values. In the case of other fragrances, the values were comparable.

### 3.2. The Physicochemical Properties of the Obtained Nanoemulsions

So far, a few methods of obtaining nanoemulsions with a low-energy method have been proposed, including spontaneous emulsification, phase inversion method, and solvent evaporation method, which perform well in the laboratory but not on an industrial scale [35]. Among the different literature reports, ultrasound homogenization is one of the most attractive methods for direct production of nanoemulsions on a larger scale [36]. However, comparative studies between scalable and non-scalable methods have not been documented enough. Even if numerous studies report the properties of nanoemulsions obtained with different methods [37], still there is no substantial knowledge about the possibility of producing a preparation optimized with a laboratory method, on a small scale, by applying a large-scale production method. This paper presents the comparison of the physicochemical properties of the optimized nanoemulsions as the media of the selected fragrance compositions obtained with a non-scalable PIC method, with systems obtained with a scalable US method.

#### 3.2.1. The Influence of a Surfactant on the Nanoemulsion Stability

In the course of the study, four hydrophilic emulsifiers were selected with an HLB (Hydrophilic-Lipophilic Balance) of 10.3–14.0, due to their very good dermatological and application properties. In order to select an optimal emulsifier, two tests were carried out—for a 6% fragrance composition concentration (corresponding to concentrations of eau de toilette, i.e., 5–15%), and for an emulsifier concentration up to 10% (not more than the traditional emulsifier systems that are safe for the skin). Table 3 presents the composition and stability of preparations obtained with the PIC and the US methods.

The data in Table 3 show that stable nanoemulsion systems were obtained with both methods, for all six fragrance compositions, with Etocas 35 (PEG-35 castor oil) only among four of the tested emulsifiers. Nanoemulsions obtained by the US method had properties similar to formulations obtained with a PIC method. Z-Ave diameter and PDI did not show any significant differences (*p* > 0.05).

According to the literature, the physicochemical properties of the nonionic emulsifiers, used for obtaining nanoemulsions, play a crucial role during emulsification. The most popular group of nonionic emulsifiers used for obtaining nanoemulsions are Tweens (ethoxylated esters of sorbitan and fatty acids) and Cremophors (ethoxylated derivatives of castor oil) [38].

Zheng et al. [38] worked on obtaining O/W nanoemulsions with a spontaneous emulsification method. On the basis of their studies, they concluded that Cremophors had better emulsification properties than Tweens. Out of the tested emulsifiers (Cremophor RH 40, Cremophor EL, Tween 20, Tween 80), nanoemulsions with the smallest droplet size and lowest PDI were obtained by stabilizing systems with Cremophor RH 40 (INCI: PEG-40 hydrogenated castor oil) and Cremophor EL (INCI: PEG-35 castor oil). The authors of the study found no correlation between the droplet size of the nanoemulsion and the HLB of the applied emulsifier. Tween 20 and Tween 80 have a similar HLB and the nanoemulsions stabilized with them had different particle sizes—179 and 122 nm, respectively. They explained the results with the impact of the molecular structure of the surfactant and, therefore, the packing parameter on emulsification. The differences between packing parameters of a surfactant affect the curvature of its monolayer on the inter-phase surface [39]. The changes of this curvature, during nanoemulsion emulsification, are crucial parameters in the process of obtaining nanoemulsions with low-energy methods (Phase Inversion Temperature (PIT); Phase Inversion Composition (PIC); Self-Emulsification (SE); Catastrophic Phase Inversion (CPI); Emulsion Phase Inversion (EPI)). 

On the other hand, Tang et al. [31] obtained nanoemulsions that were stabilized with Cremophor EL with the US method. The oil phase was formed by propylene glycol monolaurate. Nanoformulations were characterized with kinetic stability and droplet size under 200 nm. It was observed that the optimal surfactant concentration for the greatest dispersion rate of the systems was in the range of 5–6%. The authors of the research supposed that a significant decrease in the droplet size could be attributed to the presence of the polar covalent parts in the Cremophor EL chemical structure (polyethylene glycols and ethoxylated glycerol). These two components have good properties to solubilize various essential oils and hydrophobic drugs. Due to these two highly hydrophilic groups in the aqueous phase, the viscosity difference between the two immiscible phases is reduced, leading to a decrease in the critical Weber number, followed by an increased droplet break-up efficiency. The good stability of nanoemulsion droplets can be attributed to the steric stabilizing effect of nonionic emulsifier (Cremophore EL), in which it prevents flocculation and coalescence, by forming a thick steric barrier against droplet merging.

Moreover, many other studies showed that Cremophor EL was perfect to obtain nanoemulsion systems [10,40,41,42,43].

#### 3.2.2. The Effect of the Surfactant:Oil (S:O) Mass Ratio on Nanoemulsion Properties

In the next stage of the study, the emulsifier concentration was reduced to 6%, while maintaining the same fragrance composition concentration (6%) (S:O ratio = 0.5:0.5). Attempts were also made to increase the fragrance composition concentration to 15% (corresponding to the composition concentrations in eau de parfum), while maintaining the surfactant concentration at 10% (S:O ratio = 0.4:0.6). Table 4 shows the results of the analysis of particle size and PDI for three different surfactant:oil (S:O) mass ratios on the day of obtaining the preparation.

As can be seen from the data in Table 4, all fragrances (A–F) achieved satisfactory values with reduced emulsifier concentration (S:O = 0.5:0.5) and with increased fragrance composition concentration (S:O = 0.4:0.6) The droplet size did not exceed 150 nm and the PDI was not greater than 0.5, in most cases. However, increasing the fragrance composition concentration (S:O = 0.4:0.6) resulted in increasing the average droplet size of the nanoemulsion and PDI, which is consistent with the literature data, according to which the droplet size increases with the concentration of the inner phase [10,40,44,45,46]. On the other hand, the systems in which the surfactant concentration was the highest (S:O = 0.625:0.375), showed significantly larger dispersion rate. As reported in the literature, high surfactant concentration can cause complete dissolution of the oil phase in water, at the emulsion inversion point, but low surfactant concentration resulted in the formation of larger droplets. When surfactant:oil ratio (S:O) was high enough, the surfactant formed the layer structure at phase inversion point, and then the minimal interfacial tension was formed and promoted the formation of small droplets [18].

#### 3.2.3. The effect of the Fragrance Compositions on Nanoemulsion Properties

**Figure 1:**
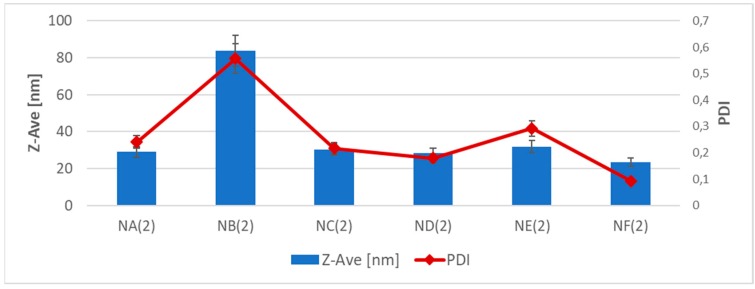
Effect of a fragrance composition on the particle size and polydispersity index (PDI) of the nanoemulsion (S:O = 0.5:0.5) (SD, *n* = 3).

With regards to the effect of the type of composition on the physicochemical properties of the nanoformulation (Figure 1, Table 4), Composition B with the highest lipophilicity (logP = 4.35, Table 2), surface tension (τ = 30.7 mN/m, Table 2), and viscosity coefficient (η = 150 mPa·s, Table 2) yielded a nanoemulsion with the largest droplet size and the highest PDI (for S:O = 0.5:0.5 and 0.4:0.6). In the case of other Compositions A, C, D, E, and F (Table 4, Figure 1), no major differences were observed between the droplets of the inner phase.

According to the literature, there is no clear relationship between the size of the nanoemulsion droplets and physicochemical properties of the oil phase (refractive index, density, interfacial tension, and viscosity). Instead, the phase behavior of the surfactant-oil-water system is likely to be more important [44,47]. 

The effect of the oil phase on the nanoemulsion droplet size obtained with a low-energy method (EPI), was studied by Ostertag et al. [47]. The systems were stabilized with emulsifiers from oxyethylated esters of sorbitan and fatty acids (Tween 20, Tween 80, Tween 85). On the basis of the conducted experiments, the researchers found out that the smallest nanoemulsion droplet sizes were obtained with medium-chain triglycerides, then with essential oils (orange and limonene), and the largest droplets when the oil phase consisted of natural oils with long-chain triglycerides (olive, grape, sesame, peanut, and canola oils). Out of the natural oils, the nanoemulsions with the smallest droplet size of the inner phase were obtained with olive, and the largest ones with canola oil.

Similar studies were carried out by Kmaiko and McClements [44], with the same types of oil phases and surfactants. The difference lay in that in order to receive the nanoemulsion, a different low-energy method—spontaneous emulsification (SE)—was used. Additionally, in this case, the droplet size followed the order—medium chain triglycerides < flavor oils < long-chain triglycerides. However, in contrast to the observations made by Ostertaga et al. [47], the nanoemulsions made on the basis of olive oil had the lowest dispersion index.

As was already mentioned, the literature reports on nanoemulsion, as a matrix for fragrance composition, concern mostly the emulsification of a single lipophillic component, mainly D-limonene. [18,23,24]. Li et al. [18] obtained nanoemulsions with the CPI (Catastrophic Phase Inversion) method, on the basis of olive oil as a carrier of D-limonene, stabilized with Tween 80. The nanoemulsions were obtained with a maximum 15% concentration of olive oil. Similar results were achieved when corn oil, sunflower oil, and soybean oil were used instead of olive oil. However, the obtained systems differed in droplet sizes (the smallest in the case of olive oil with 15% concentration of oil phase). Moreover, the nanoemulsions containing only olive oil had larger sizes than those which also had D-limonene in the oil phase. The authors explained this phenomenon with the differences in the viscosity of the oil phase. D-limonene was characterized by a much lower viscosity (approx. 2 mPa·s) than the vegetable oils (above 30 mPa·s). The viscosity of the oil phase could affect the rate of mass transport of surfactant molecules through the oil and into the aqueous phase or droplet disruption, within a flow field [47].

The nanoemulsion in which the oil phase was formed only with D-limonene was obtained by Li and Chiang [23], with the US method. The system was stabilized with a surfactant mixture—sorbitane trioleate and polyoxyethylene (20) oleyl ether. The ultrasound power was 18 W, sonification time 100–140 s. Nanoemulsions had a droplet size under 100 nm and maintained kinetic stability for eight weeks.

#### 3.2.4. The Kinetic Stability of the Obtained Nanoemulsions

**Figure 2:**
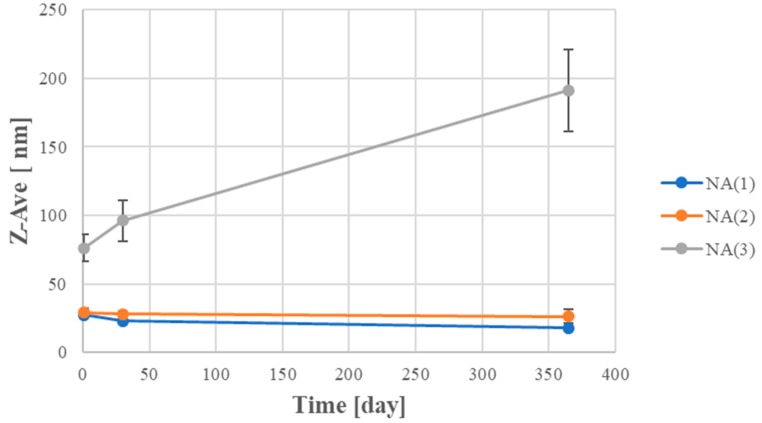
Particle size distribution over time for nanoemulsions based on fragrance A with different mass ratios of the surfactant:oil (NA(1) = 0.625:0.374; NA(2) = 0.5:0.5; NA(3) = 0.4:0.6) (SD, *n* =3).

The kinetic stability of the obtained fragrance composition nanocarriers was studied by measuring droplet sizes and PDI for nanoemulsions stored for one year, at ambient temperature. The results of the measurements are presented in the Figure 2.

In Figure 2, it can be clearly seen that the nanoemulsions on the basis of the fragrance Composition A, in which the surfactant:oil ratio (S:O) was 0.625:0375 and 0.5:0.5, maintained kinetic stability for 12 months. However, in the case of preparations with higher content of oil phase, the droplet size increased significantly, most likely due to coalescence and Ostwald ripening. 

**Figure 3:**
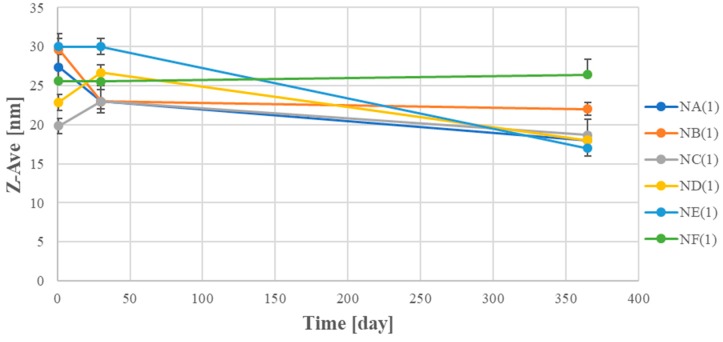
Droplet sizes of the nanoemulsions on the basis of A–F compositions as a time function (S:O = 0.625: 0.375) (SD, *n* = 3).

**Figure 4:**
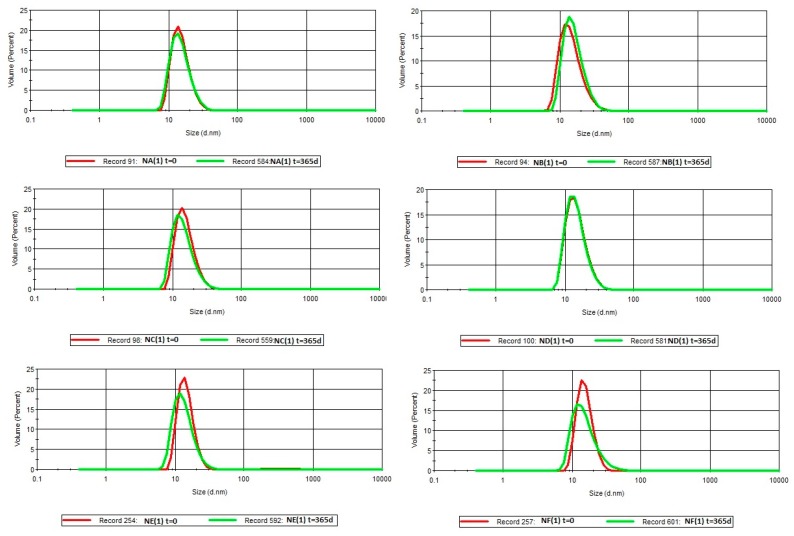
Droplet size distribution of the nanoemulsions on the basis of A–F fragrance composition, over time, for S:O = 0.625:0.375 (*t* = 0 and *t* = 365 days).

It should be noted that nanoemulsions with oil phase and surfactant in mass ratio surfactant:oil (S:O) of 0.625:0375, maintained kinetic stability in all used fragrance compositions for 12 months of storage (Figure 3 and Figure 4). The droplet sizes did not exceed 30 nm and virtually did not change, over 365 days.

These results are consistent with the literature data. Sadurni et al. [40] obtained nanoemulsions also stabilized with Cremophor EL (PEG-35 castor oil), on the basis of caprylic and capric triglycerides (Miglyol 812), with a low-energy method. For a water/Cremophor EL/Miglyol 812 system, the nanoemulsion was obtained with an oil:surfactant ratio of 10:90 to 40:60. They had a droplet size between 14 and 39 nm and maintained kinetic stability for 7 months.

#### 3.2.5. The Physicochemical Properties of the Obtained Nanoemulsions

In the next stage of the research, the physicochemical properties were established for the stable nanoformulations containing Etocas 35 emulsifier. Viscosity, pH, surface tension, and density were measured. The results are shown in Table 5.

Table 5 shows that the obtained stable preparations had pH values, appropriate for the skin [48,49], low viscosity, density, and surface tension, which would facilitate the application of nano-perfumes as aerosol. The method used to obtain the composition with the above-mentioned content, did not have any effect on the properties of the preparations. Nanoemulsion obtained by US method had properties similar to those of formulations obtained with a low-energy method. Z-Ave diameter, PDI, viscosity, surface tension, pH, and density values did not show any significant differences (*p* > 0.05).

#### 3.2.6. Oxidative Stress Test

**Figure 5:**
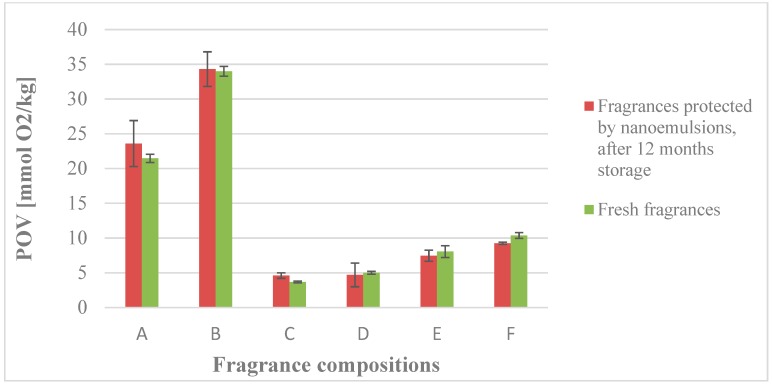
The peroxide values of the fragrances protected by nanoemulsions, after 12 months storage, and the fresh fragrance compositions (SD, *n* = 3).

In order to confirm the effect of the nanoemulsions on the chemical stability of fragrance compositions, an oxidative stress test was carried out. The peroxide number measurement (POV) helped to determine the chemical stability of the applied fragrances. The same amount of the oils (fragrance compositions) was used in all of the formulations. The calculated values of the peroxide number (POV) indicated the level of the oil oxidation process. The lower were the peroxide number differences between the fresh fragrances and those protected by nanoemulsions during storage, the higher was the protective ability of the nanoemulsion and chemical stability of the oils. Figure 5 shows the protection effect of nanoemulsion against oxidation of the oils. The obtained formulations showed a high protection against the oxidation of fragrance compositions, compared to the reference samples of pure oils. This protective ability is connected with the nanoemulsion structure, which hinders the oxygen from reaching the oils, and oxidizing them.

#### 3.2.7. The Results of the Test of Fragrance Permanence in the Nanoemulsions

The test of fragrance permanence was carried out according to the standard BN-84/6148-02 by an organoleptical assessment of the intensity of the fragrance after 1, 24, and 48 h, after dipping the filter paper in the tested formulation. The samples were assessed by comparing the intensity of the fragrance of the tested sample and the reference sample. In all cases, the intensity was the same.

#### 3.2.8. The Results of the Test of Microbiological Properties of the Nanoemulsions

The results of the microbiological tests confirmed that the obtained products were free of microbiological contamination. The total number of aerobic mesophilic microorganisms, as well as the number of yeast and mold, was below 10 cfu. The tested samples were free of pathogens, such as *Staphylococcus aureus, Pseudomonas aeruginosa*, *Escherichia coli*, and the *Candida albicans* yeast. The tests of the preservation method showed that it fulfilled the standard requirements, acceptance criteria lgRx (logarithm of the reduction of the number of viable microorganisms); in all cases it had the value of <2. It should be noted that the microbiological stabilization of the preparations did not involve any of the compounds from the list of preservatives allowed in cosmetic products presented in the Annex V of the Regulation (EC) No. 1223/2009. The raw materials used were sodium salt of levulinic acid and sodium salt of anisic acid, which are substances of natural origin that are used in cosmetics as ingredients of fragrance compositions and alternative preservatives. 

#### 3.2.9. The Results of the Test of the Dermatological Properties of the Nanoemulsions

The dermatological test for the obtained products was conducted with a patch test on a group of twenty-five testers (15 women and 10 men, age 19–55), under a dermatologist’s supervision. The assessment was carried out in accordance with a generally-accepted scale for determination of various dermatological changes (Table 6).

On the basis of the obtained results, it was found out that the tested products did not cause any side effect symptoms, irritation, or sensitization in the test group. The patch tests for all subjects gave a negative result (no reaction), therefore, neither skin irritation nor sensitization was observed as a result of the application of the preparation. It should be noted that this opinion does not apply to people who have an allergy to any of the components of the tested cosmetic.

## 4. Conclusions

As a result of the performed studies, stable oil-in-water (O/W) nanoemulsions were obtained, which were compatible with the six selected fragrance compositions, and they did not contain ethanol or any other solvent. These systems can be successfully applied as modern carriers of selected fragrance compositions, both with a low-energy method (PIC), at a laboratory scale, and with a high-energy method (US), at a production scale. The optimized nano-perfume recipes that were obtained with different methods, yielded the same physicochemical properties (stability, medium droplet size of the inner phase, polydispersity, viscosity, surface tension, pH, and density). The simple composition of the formulation is worth noting. The use of a nonionic surfactant that was gentle to the skin as an emulsifier, i.e., castor oil (Etocas 35) ethoxylated with 35 moles of ethylene oxide, allowed to obtain stable systems, without the need to use additional solubilizers, such as polyol, e.g. glycerin, an oil phase component, or an oil with low polarity (e.g., isohexadecane). Stable, transparent nano-perfumes were obtained with a fragrance composition concentration within 6–15% range [50,51]. These formulations have low viscosity and pH suitable for the skin. Moreover, the obtained results confirmed the protective role of nanoemulsions. The peroxide number measurement (POV) showed that the tested fragrance compositions had a high chemical stability. The results of the microbiological tests confirmed that the obtained products were free of microbiological contamination and were appropriately preserved. The dermatological test results confirmed the safety of the developed preparations.

## 5. Patents

- P.426105: patent application, Sikora, E.; Miastkowska, M.; Lason, E.; Gut, K.; “Method of making non-alcoholic perfumes”, application for granting a patent adopted in the Patent office of the Republic of Poland on 28.06.2018, unpublished yet.
- P.426104, patent application, Sikora, E.; Miastkowska, M.; Lason, E.; Gut, K.; “Non-alcoholic perfumes and the method of making non-alcoholic perfumes”, application for a patent granted in the Patent office of the Republic of Poland on 28.06.2018, unpublished yet.

## Figures and Tables

**Table 1:** Emulsifiers used in the research.

No.	Polish Name/Trade Name	INCI Name	HLB
1.	Cithrol 10GTIS	PEG-20 Glyceryl Triisostearate	10.3
2.	Decyl Glucoside	Decyl Glucoside	13.0–14.0
3.	Natragem S140	Polyglyceryl-4 Laurate/Sebacate (and) Polyglyceryl-6 Caprylate/Caprate (and) Water	14.0
4.	Etocas 35	PEG-35 Castor Oil	13.0

**Table 2:** Physicochemical properties of fragrance compositions.

Name of the Fragrance Composition	logP	τ (mN/m)	η (mPa·s) for γ = 1/100 s
A	2.92	23.3	123
B	4.35	30.7	150
C	3.12	25.1	108
D	3.48	24.8	102
E	3.75	26.1	140
F	3.25	26.4	129

**Table 3:** The influence of a fragrance composition (O) and emulsifiers (S) on the physicochemical properties of nanoemulsions (ratio S:O = 0.625:0.375) obtained with both methods.

Emulsifier	Fragrance Composition	Appearance	Physicochemical Properties		
			Z-Ave (nm) (*n* = ±S.D.)	PDI (*n* = ±S.D.)	Stability After 24 h
Cithrol 10GTIS	A	Milky	-	-	-
	B	Milky	-	-	-
	C	Milky	-	-	-
	D	Milky	-	-	-
	E	Milky	-	-	-
	F	Milky	-	-	-
Decyl Glucoside	A	Milky	-	-	-
	B	Milky	-	-	-
	C	Milky	-	-	-
	D	Transparent	21.0 ± 0.2	0.269 ± 0.013	+
	E	Milky	-	-	-
	F	Milky	-	-	-
Natragem S140	A	Semi-transparent, bluish	123 ± 15	0.750 ± 0.008	+
	B	Milky	-	-	-
	C	Semi-transparent, bluish	106 ± 20	0.692 ± 0.066	+
	D	Semi-transparent, bluish	151 ± 44	0.633 ± 0.002	+
	E	Semi-transparent, bluish	120 ± 10	0.592 ± 0.066	-
	F	Semi-transparent, bluish	105 ± 14	0.433 ± 0.002	+
Etocas 35	A	Transparent, bluish	27.2 ± 0.9	0.509 ± 0.061	+
	B	Transparent, bluish	23.5 ± 0.4	0.308 ± 0.007	+
	C	Transparent, bluish	19.8 ± 0.2	0.214 ± 0.002	+
	D	Transparent, bluish	22.0 ± 0.3	0.346 ± 0.007	+
	E	Transparent, bluish	30 ± 2	0.467 ± 0.060	+
	F	Transparent, bluish	25 ± 1	0.537 ± 0.012	+

- unstable sample (separation); + stable sample (homogeneous).

**Table 4:** The effect of the mass ratio of the surfactant—Etocas 35 (S)—in relation to fragrance composition (O) on the particle size (Z-Ave) and polydispersity index (PDI) of the obtained nanoemulsions.

Composition	S:O		
	(1)—0.625:0.375	(2)—0.5:0.5	(3)—0.4:0.6
	Z-Ave (nm)/PDI (*n* = ±S.D.)		
A	27.2 ± 0.9/0.509 ± 0.061	29.1 ± 0.1/0.241 ± 0.021	96 ± 20/0.536 ± 0.059
B	23.5 ± 0.4/0.308 ± 0.007	84 ± 14/0.557 ± 0.019	133 ± 5/0.471 ±0.013
C	19.8 ± 0.2/0.214 ± 0.002	30.2 ± 0.4/0.217 ± 0.016	88 ± 5/0.497 ± 0.085
D	22.0 ± 0.3/0.346 ± 0.007	28.0 ± 0.4/0.179 ± 0.009	62 ± 1/0.416 ± 0.004
E	30 ± 2/0.467 ± 0.060	32 ± 2/0.292 ± 0.012	40.3 ± 0.6/0.286 ± 0.006
F	25 ± 1/0.537 ± 0.012	23.0 ± 0.3/0.093 ± 0.009	41.2 ± 1.1/0.345 ± 0.029
Legend: (1)—surfactant:oil (S:O) mass ratio = 0.625:0.375; (2)—surfactant:oil (S:O) mass ratio = 0.5:0.5; (3)—surfactant:oil (S:O) mass ratio = 0.4:0.6.			

**Table 5:** Physicochemical properties of nanoemulsions containing different fragrance compositions with an S:O ratio of 0.625:0.375 (SD, *n* = 3).

Name of the Sample	Z-Ave (nm)	pH	Viscosity Coefficient (mPa·s), γ = 100 s^−1^	Surface Tension (mN/m)	Density (g/cm^3^)
NA(1)	27.2 ± 0.9	4.95 ± 0.2	78.2 ± 5.0	32.1 ± 1.5	1.005 ± 0.001
NB(1)	23.5 ± 0.4	5.28 ± 0.3	70.4 ± 2.5	35.6 ± 2.0	1.007 ± 0.003
NC(1)	19.8 ± 0.2	5.05 ± 0.15	69.5 ± 9.0	33.6 ± 1.4	1.008 ± 0.002
ND(1)	22.0 ± 0.3	5.08 ± 0.10	70 ± 4	33.2 ± 2.0	1.006 ± 0.003
NE(1)	30 ± 2	4.87 ± 0.25	71 ± 7	33.5 ± 0.9	1.007 ± 0.003
NF(1)	25 ± 1	4.47 ± 0.18	69.8 ± 9.6	33.5 ± 1.0	1.007 ± 0.005

**Table 6:** Skin assessment by a dermatologist.

Designation	Type of Change
(-)	No reaction
(+/-)	Weak short-term itching
(+)	Weak itching and local weak erythema
(++)	Itching and local erythema
(+++)	Itching, large erythema and papules
